# Long-Term Follow-Up of Computer-Assisted Microvascular Mandibular Reconstruction: A Retrospective Study

**DOI:** 10.3390/jcm13133899

**Published:** 2024-07-03

**Authors:** Erika Crosetti, Pierluigi Tos, Mattia Berrone, Bruno Battiston, Giulia Arrigoni, Giovanni Succo

**Affiliations:** 1 ENT Clinic—Head and Neck Cancer Unit, San Giovanni Bosco Hospital, 10154 Turin, Italy; erika.crosetti@aslcittaditorino.it (E.C.); m_berrone@hotmail.com (M.B.); giulia.arrigoni@aslcittaditorino.it (G.A.); giovanni.succo@unito.it (G.S.); 2 Hand Surgery—Reconstructive Microsurgery Clinic, ASST Centro Specialistico Ortopedico Traumatologico Gaetano Pini-CTO, 20122 Milan, Italy; 3 Hand Surgery and Reconstructive Microsurgery Department, CTO Hospital, A.O.U. Città della Salute e della Scienza, 10126 Turin, Italy; bruno.battiston@virgilio.it; 4 Department of Oncology, University of Turin, 10127 Turin, Italy

**Keywords:** mandible, mandibular reconstruction, head and neck squamous cell carcinomas, planning technique

## Abstract

**Background:** Virtual surgical planning has become a well-established practice in head and neck surgery. In oncological surgery, it permits the achievement of safe margins resections and ensures functional reconstructions and optimal esthetic outcomes. This study aimed to evaluate the long-term outcomes after virtually planned mandibular microvascular reconstruction, focusing on functional and esthetic results, as well as health-related quality of life. **Methods:** A long-term retrospective evaluation of 17 patients with oral cavity malignancy who underwent computer-assisted mandibular resection and reconstruction was performed. Functional and esthetic outcomes were analyzed using the EORTC, QLQ-C30, H&N35, and FACE-Q questionnaires. **Results:** Time since reconstruction ranged from 7 to 14 years. Patients reported high functional levels on the QLQ-C30 functional scales but lower scores on H&N35. On FACE-Q, patients demonstrated higher appraisal and satisfaction with their smiles compared to their overall facial appearance. **Conclusions:** In this retrospective case series, patients undergoing computer-assisted mandibular reconstruction for oral malignancies achieved good long-term functional and esthetic outcomes. Although limited by the small sample size, these results support the enduring benefits of virtual planning for mandibular reconstruction. To minimize declines in function and appearance, considerations should include immediate dental implants, enhanced reconstruction of the temporomandibular joint, newer methods of radiotherapy to minimize xerostomia, and oral exercises to prevent trismus.

## 1. Introduction

In the domain of head and neck oncology, particularly focusing on oral cavity squamous cell carcinoma (OCSCC), surgical intervention is considered the primary therapeutic option. The traditional metrics for evaluating surgical success have centered around short-term outcomes such as surgical efficacy, oncological radicality, and patient survival. However, recent advances in medical technology and the growing need to manage healthcare expenditures have brought to light the often-neglected aspect of patient-centered outcomes like health-related quality of life (HRQOL). This aspect is particularly vital for patients living with chronic, nonlethal conditions or those expected to survive many years after diagnosis, as even minor improvements in HRQOL can significantly impact both the patients and their families, thereby influencing the choice of treatment to ensure a balance between longevity and quality of life.

Moreover, the complexity of treating OCSCC is exacerbated by the oral cavity’s complex three-dimensional anatomy, requiring surgical precision to ensure negative margins of at least 1 cm in all directions, when possible. When OCSCC involves the mandible, either directly or through the contiguous spread, it often necessitates either marginal or segmental mandibular resection. Historically, mandible reconstruction was not always prioritized, leading to significant esthetic deformity and compromised oral function. Attempts at reconstruction using metallic plates or nonvascularized bone grafts frequently resulted in unsatisfactory outcomes [1,2,3,4,5]. 

The advent of microsurgery and vascularized free flaps has revolutionized reconstructive surgery in head and neck oncology. The fibula free flap (FFF), initially introduced by Taylor and popularized by Hidalgo, is now widely considered the gold standard for reconstructing the mandible [6,7,8]. Such reconstructions not only improve the patient’s remaining quality of life but also prevent the need for more extensive secondary reconstructive surgeries and frequently allow for the use of fixed dental prostheses. The innovation of virtual resection/reconstruction planning, especially computer-assisted mandibular reconstruction (CAMR), has significantly enhanced this field by markedly shortening surgery times and achieving unprecedented precision in modeling the revascularized bone to replace the mandible, improving functional and esthetic outcomes and reducing the rate of complications [9,10,11,12,13,14,15,16].

Although CAMR with FFF achieves promising immediate esthetic outcomes in mandibular reconstruction, the long-term picture remains less clear. Several factors contribute to this knowledge gap that should be more thoroughly analyzed: firstly, the long-term recovery of oral functions; secondly, the impact of bone and soft tissue atrophy on both esthetics and function, particularly after radiotherapy or chemoradiotherapy. Radiotherapy and chemoradiotherapy are known to compromise tissue vascularity, leading to long-term bone resorption and soft tissue shrinkage. These can negatively affect implant stability, facial symmetry, and ultimately patient quality of life.

Finally, the natural aging process adds another layer of complexity. The age-related declines in bone density and elasticity can further exacerbate the esthetic deterioration observed in long-term cancer survivors who underwent CAMR.

Therefore, there is a critical need for well-designed longitudinal studies to comprehensively evaluate long-term outcomes following CAMR with fibula free flaps.

The aim of this study was to assess the long-term outcomes, in terms of functional and esthetic results as well as HRQOL, in patients who underwent virtually planned mandibular microvascular reconstruction 7 to 14 years ago. This comprehensive approach aimed to underscore the integration of technological advancements in surgical planning with patient-centered outcomes, thereby redefining success metrics in the treatment of OCSCC to equally prioritize survival, HRQOL, and surgical precision.

## 2. Materials and Methods

A retrospective chart review was conducted on 17 long-surviving patients who had advanced oral cavity malignancies and underwent segmental mandibulectomy with virtual mandibular FFF reconstruction between 2010 and 2017. All procedures were conformed to conventional techniques and indications, in accordance with current guidelines and the ethical standards established by the Institutional and/or National Research Committee, along with the 1964 Helsinki Declaration and its later amendments. Patients were thoroughly briefed on the advantages and disadvantages of the treatment and the available alternatives during the process of obtaining informed consent. Four surgeons (G.S., E.C., P.T., B.B.) carried out all the procedures (2 head and neck surgeons performed the mandibular resection and reconstruction, 2 orthopedic/plastic surgeons carried out the FFF harvesting). One dentistry colleague (M.B.) (prosthodontist) performed the preoperative dental evaluation and the postoperative dental rehabilitation. The demographic information of the series is provided in Table 1.

Every patient underwent segmental mandibulectomy. The resections and reconstructions were meticulously planned virtually, and surgeries were facilitated by the use of bone-cutting guides. The strategy for bone resections aimed to ensure a minimum margin of 1 cm from the radiologically visible lesions. Furthermore, additional clinical evaluations during the virtual planning process aimed to reduce the risk of underestimating tumor spread within perimandibular tissues. A computer-assisted mandibular reconstruction (CAMR) program was employed for virtual resection and reconstruction, utilizing specialized software to facilitate the creation of custom cutting guides and plates via CAD-CAM technology.

Mandibular reconstruction affects multiple aspects of a patient’s life: physical function (eating, speaking), emotional well-being (self-image), and social interaction. Using a single questionnaire might miss a crucial dimension; therefore, the quality of life (QL) and functional outcomes for patients were assessed using the European Organization for Research and Treatment of Cancer (EORTC) core Quality of Life Questionnaire (EORTC QLQ-C30, version 3) and its head and neck cancer-specific module (EORTC QLQ-H&N35) [17,18]. These instruments have shown excellent acceptability, internal consistency, reliability, test–retest reliability, and high responsiveness, as well as good convergent validity with the University of Washington Quality of Life questionnaire (UWQOL), addressing many critical aspects of the head and neck cancer patient experience. They have been thoroughly developed and validated, offering robust alternatives to the UWQOL. Furthermore, the FACE-Q Oncology Module: Mandibulectomy, an innovative patient-reported outcome (PRO) measure, was utilized to assess patients’ perceptions of their esthetic outcomes. This tool includes subscales related to function, enhancing the functional insights provided by the EORTC evaluations. Specifically, four FACE-Q scales [19,20] were employed to evaluate patients’ esthetic outcomes concerning (a) their assessment of their smile, (b) their satisfaction with their smile, (c) their assessment of their facial appearance, and (d) their satisfaction with their facial appearance. This comprehensive approach ensured a nuanced understanding of the impact of treatment on patients’ quality of life and functionality, highlighting the significance of both clinical outcomes and patient-reported measures in the holistic care of individuals with head and neck cancer.

Continuous variables are reported as mean (SD) and categorical variables as frequencies and percentages. Correlations between variables were evaluated with Pearson’s test. A two-sided *p* value < 0.05 was considered statistically significant. All analyses were performed with SPSS 20.0 (IBM corp., Armonk, NY, USA).

## 3. Results

A total of 17 patients (10 men and 7 women) with an overall average age of 59 years at the time of surgery (men: age range, 33–78; women: age range, 43–78 years) affected by advanced oral cavity malignant tumors with bone and/or perimandibular soft tissue and periosteal invasion were surgically treated. Six patients had received prior treatment (five patients by transoral surgery, two patients by multimodal therapy (surgery + radiation therapy). 

OCSCC was confirmed in 15 patients (88.2%); in 1 patient, a verrucous carcinoma was diagnosed; and, in another, a mandibular metastasis from breast cancer was diagnosed.

Each patient was reviewed by the multidisciplinary team, and, for 15 patients, a recommendation was made for postoperative chemoradiotherapy.

The time since reconstruction ranged from 7 to 14 years.

Only nine patients (52.9%) opted for secondary dental implant rehabilitation.

All patients maintained regular mandibular movement with physiological sliding after surgery, and no mandibular head dislocation was observed. 

Various questionnaires were utilized to assess the different aspects of QoL. Employing multiple validated questionnaires, each with recognized strengths and weaknesses, enhanced the overall quality of this study. This approach enabled triangulation, where findings from different instruments converged and supported one another.

Table 2 summarizes the results.

**Figure 1:**
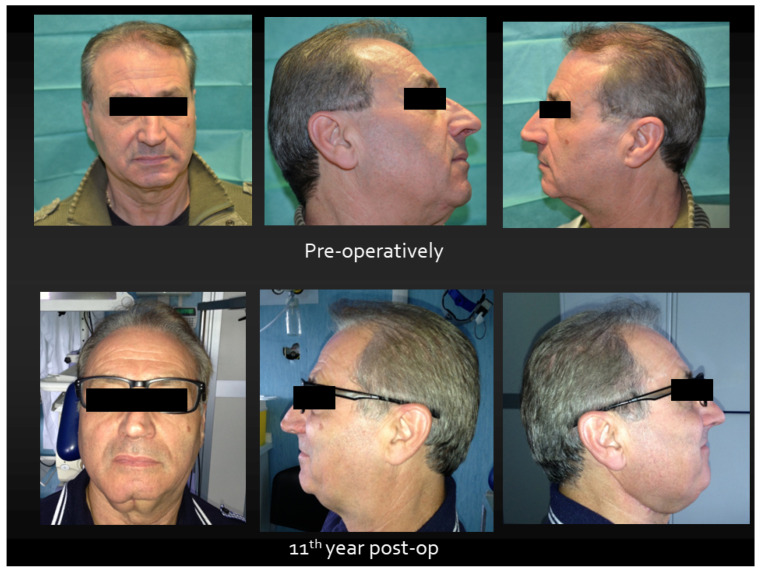
Functional and esthetic results 11th years after surgery.

**Figure 2:**
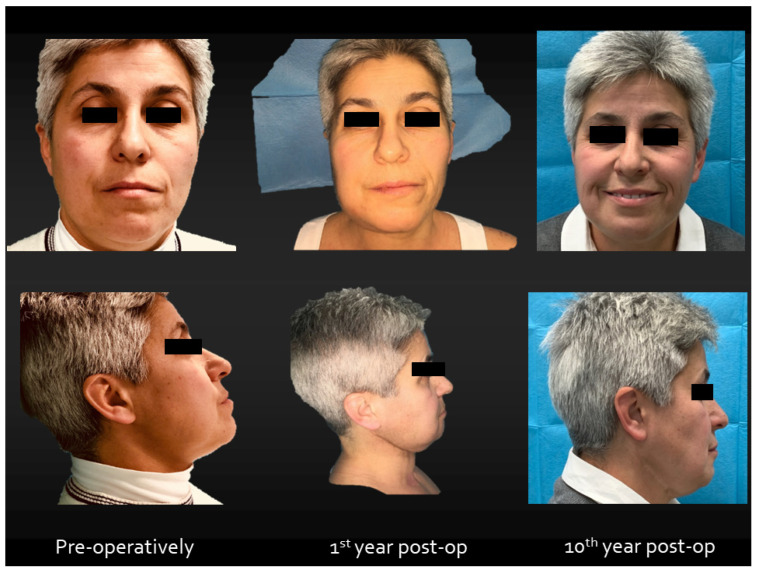
Functional and esthetic results 10th years after surgery.

On FACE-Q, patients provided a higher appraisal of and satisfaction with their smiles compared to their overall facial appearance (Figure 1 and Figure 2).

Patients reported high functional levels on the QLQ-C30 functional scales, with all mean functional scale scores exceeding 76 on a scale of 0–100. The lowest functional scores were observed for global health status/quality of life (QL mean, 71.3), while the highest scores were for physical functioning (PF mean, 92.7). General symptom scale scores on the QLQ-C30 were relatively low, with the highest scores reported for insomnia (S -mean, 23) and constipation (CO mean, 18.5), and the lowest for nausea/vomiting (NV mean, 1.5) and diarrhea (DI mean, 2.8).

The symptom scales on QLQ-H&N35, which assesses symptoms specific to head and neck cancer, showed comparatively higher scores than those on the more general QLQ-C30 symptom scales. The highest scores on QLQ-H&N35 were for dry mouth (49.5), teeth (mean, 38.9), opening mouth (38.8), less sexuality (mean, 36.0), and sticky saliva (mean, 35). The lowest scores on QLQ-H&N35 were for feeling ill (mean, 8.0) and trouble with social contact (mean, 11.7). 

Time since reconstruction significantly correlated with a more positive appraisal of smile on FACE-Q (Pearson’s r = 0.71; *p* < 0.01) and fewer sexual symptoms on QLQ-H&N35 HNSX (r = −0.59, *p* < 0.01), indicating that patients had more favorable smile appraisals and experienced fewer sexual symptoms as time passed since their reconstruction. Conversely, greater time since reconstruction was associated with worse physical functioning (r = −0.48, *p* < 0.05) and increased loss of appetite (r = 0.49, *p* < 0.05) on QLQ-C30, which may have been partly due to the normal aging process.

## 4. Discussion

Limited studies have detailed the quality of life (QL) and functional outcomes following mandibulectomy and reconstruction with fibula free flap, especially in cases in which a computer-assisted mandibular reconstruction program was employed [14,21]. It is widely recognized that as time passes and individuals age, both the general appearance of the face and specifically reconstructed tissues experience deterioration. Notably, progressive atrophy can be observed both 5 and 10 years after surgery. However, such significant atrophy typically does not occur in patients who have not received radiation therapy, aside from normal aging-related esthetic changes. Conversely, patients undergoing post-operative radiotherapy (RT) exhibit accelerated atrophic changes, which can begin as early as 2 to 4 years following RT, leading to continual declines in both function and aesthetic appearance. The normal aging process further compounds the degradation across all functional and esthetic domains, making these factors crucial in evaluating long-term survivors of FFF mandible reconstruction.

In 2018, Petrovic et al. [22] analyzed a patient cohort over an extended period (from 1987 to 2013) at a tertiary care cancer center, focusing on individuals who underwent fibula free flap reconstruction following segmental mandibulectomy for oral cancer.

Notably, while patients reported high physical function, physician evaluations based on the 11-item questionnaire suggested only a 64% achievement of ideal function. The lack of natural teeth and inadequate mastication, alongside malocclusion, were identified as key factors impacting these scores. Oral competency was another notable contributor to the functional decline observed. Previous studies [23,24,25,26,27] corroborate these findings, highlighting the challenges faced by patients with oral cancer in wearing dental prostheses, whether they underwent radiation therapy or not. The challenge of secondary dental implant placement, with only a few patients managing to complete this process for implant-supported dentures, partially explains the lower satisfaction with oral function reported by patients, reflecting the complexities and challenges in post-mandibulectomy and FFF reconstruction care.

Other factors contributing to diminished function included xerostomia in patients who had undergone radiation therapy and reduced sensation in the lower lip, leading to an average functional recovery score of 64% from clinicians. The global health status on the EORTC QLQ-C30 scale, which examines QL months to years after surgery, consistently received the lowest scores. This overall measure of QL showed that patients predominantly struggled with social disability, handicap, and psychological distress. Similar measures were assessed using the UWQOL by Yang et al., pointing to continuous challenges faced by patients regarding communication and swallowing, potentially affecting their QL.

A study by Kumar et al. [28] highlighted the benefits of implant-supported dentures over removable ones in patients undergoing dental rehabilitation after segmental mandibulectomy and FFF reconstruction. Measured at three intervals—before surgery, 6 months, and 1 year after surgery—the results indicated significantly better functional outcomes and QL with implant-supported dentures. This suggests that immediate implant placement during FFF reconstruction might be advantageous for more stable denture support, although economic and patient interest factors can present barriers.

Research, including cross-sectional and longitudinal studies on QL after mandibulectomy with free flap reconstruction, has shown that patients undergoing irradiation report a lower global QL than their nonirradiated counterparts. This disparity is likely due to RT’s negative impacts, as irradiated patients exhibit a higher incidence of head and neck symptoms. Interestingly, while patients with RT may exhibit better function and fewer symptoms on the QLQ-C30 initially, they tend to report more issues than those without RT on the QLQ-H&N35 over time.

Moreover, the significance of comorbidities in affecting QL cannot be overstated. Korflage et al. [29] noted that comorbidity significantly influences QL outcomes, with irradiated patients experiencing late RT effects like xerostomia and trismus, impacting QL even years after treatment. Terrell et al. further identified comorbidity as a critical determinant of decreased QL in patients with head and neck cancer [30].

The esthetic outcomes of reconstructions also merit attention. Posch et al. [31] found that independent evaluators rated esthetic outcomes more negatively than patients did, emphasizing discrepancies in perception. This finding aligns with the observations of Petrovic et al. [22], where nonclinician scores for esthetics were generally lower than those from clinicians, indicating a range of factors—from color mismatch to flap bulkiness—that contribute to perceived esthetic quality.

The findings of this study align with those in the existing literature. The integration virtually planned mandibular resection/reconstruction represents a significant advancement in surgical procedures, providing a valuable tool in reducing surgery time and minimizing the burden of intraoperative decisions. This method enhances the accuracy of reconstruction and increases the predictability and repeatability of surgeries. Additionally, the functional benefits, the reduction in operating time, and the high precision obtained in reconstructions, as demonstrated by the authors of previous articles, seem to justify the potential risks of encountering bone-positive margins. Moreover, it is important to emphasize that these techniques have led to exceptionally high esthetic outcomes, underscoring the substantial improvements in both form and function facilitated by virtual planning of mandibular surgeries [32].

Notably, the majority of patients in this cohort (88.2%) underwent postoperative chemoradiotherapy, which led to the predominant complaints of xerostomia and dry mouth syndrome over time. It is also noteworthy that only 52.9% patients opted for dental implants at a later stage, yet they remained satisfied with both the esthetic and functional outcomes of their treatment. This aspect can be attributed to a sense of fatigue and fear following multimodal treatments, a condition that is typically observed in these patients.

Our impression is that patients reconstructed with the CAMR technique feel less ill and less impaired in social interactions, which can be attributed overall to the greater precision in the reconstruction process.

This research has several limitations, which need to be taken into account when considering the findings reported here. The natural aging process, which can lead to deterioration in esthetic appearance and function, must be considered in all such studies. Additionally, the perception and level of satisfaction among patients can vary greatly, influenced by their individual expectations. Moreover, ideally, a much larger patient cohort would be examined to address these issues thoroughly, although such a study may not be feasible due to the progressive loss of life due to disease or other unrelated factors. Despite these limitations, we are confident that this study can provide valuable insights into the long-term esthetic and functional outcomes for patients undergoing computer-assisted mandibular fibula free flap reconstruction.

The future of CAMR is promising. Advances in 3D printing and bioprinting are set to provide even better anatomical fit, functionality, and biocompatibility. Virtual and augmented reality will transform preoperative planning through surgical simulations and real-time guidance during surgery. Artificial intelligence, by analyzing patient data and imaging, will be able to predict potential complications and recommend optimal individualized surgical approaches. These combined advancements will result in improved accuracy and fewer complications.

## 5. Conclusions

Tackling challenges such as the absence of natural teeth and malocclusion, potentially through the use of immediate dental implants during free fibula flap reconstruction, and exploring innovative long-term care approaches, including new radiation techniques, could markedly enhance patient quality of life. This highlights the importance of considering a comprehensive range of factors to improve the long-term outcomes for patients undergoing FFF reconstruction of the mandible.

These insights collectively underscore the complex and multifaceted nature of recovery and long-term results following computer-assisted mandibular reconstruction. They emphasize the benefits of immediate dental implants for better functional and esthetic outcomes, the significant impact of radiation therapy on long-term QL and esthetics, and the crucial influence of comorbidities on patient outcomes. Thoroughly addressing these elements can greatly improve the QL and satisfaction of patients on the challenging path to recovery from head and neck cancer.

## Figures and Tables

**Table 1:** Demographic data for the 17 patients in this study.

Characteristics	No. of Patients (%)
**Sex**	
Male	10 (58.8)
Female	7 (41.2)
**Risk factors**	
Nonsmoker	6 (35.3)
Previous smoker	11 (64.7)
**Pretreatment**	
Yes	6 (35.3)
No	11 (64.7)
**Site**	
Retromolar trigone	5 (29.4)
Alveolar crest	4 (23.5)
Floor of the mouth	4 (23.5)
Hemimandible	3 (17.6)
Mandibular symphysis	1 (5.8)
**Histology**	
Squamous cell carcinoma	15 (88.2)
Verrucous carcinoma	1 (5.8)
Bone metastases from breast cancer	1 (5.8)
**Pathological TNM**	
pT4aN0	4 (23.5)
pT4aN2a	1 (5.8)
pT4aN2b	2 (11.7)
pT4aN2c	1 (5.8)
pT4aN3b	3 (17.4)
ypM1	1 (5.8)
ypT4aN0	5 (29.4)

TNM: a system for classifying a malignancy: T-Tumor; N-Nodes; M-Metastasis.

**Table 2:** **FACE-Q scale, QLQ-C30 and QLQ-H&N35 results**.

	Total	Mean (95% CI)
FACE-Q		
Smile appraisal	17	67.8 (2.9 to 129.1)
Smile satisfaction	17	84.1 (75 to 94.1)
Appearance appraisal	17	54.5 (37.3 to 66.7)
Appearance satisfaction	17	69 (34.3 to 103.7)
QLQ-C30		
QL	17	71.3 (62.1 to 83.9)
PF	17	92.7 (87.1 to 98.4)
RF	17	88 (74.4 to 101.5)
EF	17	81.3 (70.1 to 91.5)
CF	17	84.5 (72.7 to 95.6)
SF	17	86.1 (73 to 99.2)
FA	17	14 (3.4 to 26.5)
NV	17	1.5 (0.5 to 2.1)
PA	17	13.7 (6.3 to 23.9)
DY	17	0
SL	17	23 (6 to 32.5)
AP	17	12 (3.2 to 19.2)
CO	17	18.5 (1.1 to 27.3)
DI	17	2.8 (−1.5 to 5.4)
FI	17	14.8 (0.6 to 29)
QLQ-H&N35		
HNPA	17	16.5 (7.3 to 25.8)
HNSW	17	16.7 (3.8 to 29.6)
HNSE	17	12.2 (2.8 to 22.5)
HNSP	17	19.8 (10.7 to 32.9)
HNSO	17	22.7 (9.5 to 38.3)
HNSC	17	11.7 (4.2 to 20)
HNSX	17	36.0 (20.9 to 58.8)
HNTE	17	38.9 (24.1 to 57.7)
HNOM	17	38.8 (22.1 to 58.7)
HNDR	17	49.5 (32.3 to 69.9)
HNSS	17	35 (17.4 to 55.2)
HNCO	17	19.1 (1.8 to 36.2)
HNFI	17	8.0 (−1.2 to 17.3)

Abbreviations: QL = global health status/QoL; PF = physical functioning; RF = role functioning; EF = emotional functioning; CF = cognitive functioning; SF = social functioning; FA = fatigue; NV = nausea and vomiting; PA = pain; DY = dyspnea; SL = insomnia; AP = appetite loss; CO = constipation; DI = diarrhea; FI = financial difficulties; HNPA = pain; HNSW = swallowing; HNSE = sense problems; HNSP = speech problems; HNSO = trouble with social eating; HNSC = trouble with social contact; HNSX = less sexuality; HNTE = teeth; HNOM = opening mouth; HNDR = dry mouth; HNSS = sticky saliva; HNCO = coughing; HNFI = Felt il.

## Data Availability

The raw data supporting the conclusions of this article will be made available by the authors on request.
